# Stochastic aspects of motor behavior and their dependence on auditory feedback in experienced cellists

**DOI:** 10.3389/fnhum.2013.00419

**Published:** 2013-07-31

**Authors:** Jessie Chen, Marjorie Woollacott, Steve Pologe, George P. Moore

**Affiliations:** ^1^ Department of Physiology and Neuroscience, New York University School of MedicineNew York, NY, USA; ^2^ Department of Human Physiology, University of OregonEugene, OR, USA; ^3^ School of Music, University of OregonEugene, OR, USA; ^4^ School of Engineering, University of Southern CaliforniaLos Angeles, CA, USA

**Keywords:** motor control, serial correlation, sensory feedback, stochastic, reciprocal movements, musical performance, martingale

## Abstract

This study aimed to investigate movement accuracy of experienced cellists, the statistical properties of their note sequences during a reciprocal task, and the degree to which these movement characteristics depend on auditory feedback. Nine experienced cellists were asked to shift alternately between two notes using only their index finger to make contact with the string and fingerboard. Shifting sequences continued for two minutes at a rate of one note per second. The task was performed under two conditions: with auditory feedback (provided by the bow) or without auditory feedback (i.e., without the use of bow). When the bow was used, subjects had no difficulty in shifting between target notes with precision and stability. Some variability was present, but notes in these sequences were generally uncorrelated. The contact data and correlations in most bowed trials resembled those expected of a *renewal process*, a process in which successive values are statistically independent and identically distributed. Without the bow, subjects lost their ability to reach the same target positions accurately; contact locations tended to drift and had a random quality, indicating that without the bow subjects were uncertain of the target location in relation to the spatial location of their fingertips. Within these unbowed sequences, finger positions were highly correlated—within and between note sequences. In some trials without the bow, the statistical correlation patterns of the sequence were consistent with the expectations of a discrete *Wiener process*. Throughout our study, computer simulations of renewal and Wiener processes enabled us to determine the types of correlations to be expected from these theoretical models. The implications of the statistical results in terms of subject behavior are discussed.

## Introduction

The motor activities of stringed instrument players, i.e., performers of instruments from the violin family, must be precisely controlled. Under normal performance conditions, an experienced musician can move rapidly, alternately and repeatedly between notes with considerable accuracy. Indeed, many stringed instrument players believe they can move from note to note even without auditory feedback, as if somehow they automatically “know” where the notes are. In this study of experienced cellists, we investigated the degree to which their accuracy in moving between notes on a single string, and the statistical properties of the note sequence locations, were dependent on auditory feedback.

In this study, a specially instrumented cello was used, allowing us to track precisely the contact location between the finger, the string and the fingerboard as subjects moved repeatedly between two alternating note locations. In some trials the performers were permitted to use the bow during the shifting sequences (providing auditory feedback); in other trials the bow was not used. As expected, when the bow was used performers had no difficulty reaching the requisite note locations with considerable precision (though there was, of course, a small random—but usually imperceptible—pitch/location error). Without the bow subjects shifted between the same two target locations, but appeared to have lost any clear sense of the finger contact location in relation to the intended target locations. These errors would have been easily perceived if the bow had been used. The properties of the dual note sequences were analyzed statistically and are the main focus of this report. In trials using the bow, the note sequences most often had the statistical characteristics of an *alternating renewal process* (Cox and Lewis, 1966). Without the bow some note sequences had the characteristics of a discrete *Wiener process*, a theoretical random-walk process related to Brownian motion (Einstein, 1956; Bharucha-Reid, 1960; Chatfield, 1975). This result seems to contradict the assertion by many musicians that in the absence of auditory feedback, years of practice and performance would still enable them to reach any target note with precision. The random characteristics of sequences generated without use of the bow may actually reflect a rational attempt (a “martingale strategy”) by the performer to return alternately to the same previous target locations. This rational strategy may, paradoxically, lead to a stochastic—“random walk”—performance. We are accustomed to thinking of random walk processes as being associated with microscopic features of biological systems: diffusion and Brownian motion are two examples (Berg, 1993). But the present study shows that classic random walk processes can arise at the macroscopic—behavioral—level as well. We discuss below the connections of our study—and its results—to an existing body of research that has led to several major motor control theories.

## Methods

### Laboratory apparatus

All subjects used a cello equipped with a string circuit that measured the contact position between their first finger and the fingerboard. This allowed us to determine their finger position when they held a note and when they shifted between notes. It was described previously in Chen et al. (2006, 2008). The circuit output was digitized at 360 samples/sec. The circuit effectively measures the distance between the cello bridge and the point where the first finger makes contact between the string and the fingerboard; the contact location can be determined to within a millimeter. The distance to the bridge determines the length of the freely vibrating string and the fundamental resulting frequency of the vibration (pitch). Surrounding each exact note position is a distance within which it is difficult to detect whether the note is at the required pitch. This region, called here the “pitch window”, is one-eighth of the distance to the next higher and lower note and is wider for lower-pitched notes (being of the order of a cm or more for notes farthest from the bridge), but only a millimeter or less for notes closer to the bridge. That is, considerably greater spatial precision is required for higher-pitched notes close to the bridge. It is useful therefore to express variability of finger positions for a given note in relation to the length of its pitch window. Our pitch window calculations here are based on a string length of 68.5 cm.

### Subjects

A group of nine cellists, four males and five females, participated in this study. Seven were recruited from the University of Oregon School of Music. Two other subjects were professional cellists. Significantly, none of the subjects had absolute pitch, which means that none of them could reliably and consistently produce or identify a named note from an auditory cue.

### Protocol

Participants were asked to shift alternately between two notes on the cello A string without using vibrato at the rate of 1 note/sec for 2 minutes. Two pairs of notes were used: B (246.9 Hz) and D (293.7 Hz), and B and A (440 Hz). These pairs were separated on the fingerboard by approximately 10 and 26 cm, respectively. Each note was played using only the index finger; thus required a shifting movement of the hand and arm along the string. All subjects employed a legato bowing style and played one bow per note. The string was tuned by each subject at the beginning of each trial, using a conventional musician’s frequency meter, independently calibrated. All trials were paced by a metronome for a few seconds before data collection began; the metronome was set to one beat per second. Each trial yielded between 35 and 45 notes for each note in the pair. In an equal number of trials subjects were instructed to shift between these same note pairs but without using the bow. String vibrations were muffled to eliminate any auditory feedback. Participants were required to close their eyes during these trials, though even when playing freely most subjects did not use vision consistently to guide their movements. Bowing conditions and note pairs were randomized between trials.

For each note we determined the *modal contact position* (See Chen et al., 2008). Though subjects using the bow typically make small adjustments in contact position during each note (presumably to make small pitch corrections), we took from each note that value of the contact point assumed most of the time while the note was held. That modal value is used here in our graphic displays and subsequent calculations. In the Figures below we plot the modal value of the contact position against the serial number of the note, that number becoming an index of the sequence (replacing the time variable). Players played in tempo with a high degree of consistency so that plotting modal location against serial number is approximately equivalent to a time plot. Here, of course, time is not the variable of interest.

### Data Selection

When a subject did not use the bow we found, unexpectedly, that the shifting movements between the same notes were quite different in their details. Many subjects had less contact with the fingerboard during their silent shifts; the moment of contact for each succeeding note was therefore less precisely defined, as was the modal value. For this study we therefore used only those no-bow trials where the performer maintained sufficient contact with the string during and after the shift to make the determination of modal value unambiguous.

### Serial Calculations

For each trial, we calculated the serial auto-correlation coefficients of positions for note *x* and note *y*, and the serial cross-correlation between note *x* and note *y* positions. We also calculated the serial correlation coefficient between any note and the *k*-th note following (or preceding), calculating the correlations beyond adjacent notes. In each trial, note B was assigned as the reference note; thus, the note *y* following immediately has the same index number (*lag* = 0).

The *k*-th order correlation coefficient is computed as:

$$R(k)=\sum_{i}\left[\left(x_{i}-{\bar{u}}_{x}\right)\left(y_{i+k}-{\bar{u}}_{y}\right)/\left(\sigma_{x}\right)\left(\sigma_{y}\right)\right]$$

where *R(k)* is the *k*-th order serial cross-correlation coefficient, *ū_x_* and *ū_y_* are the mean positions of notes *x* and *y*, and *σ_x_* and *σ_y_* are their standard deviations. The corresponding auto-correlations have only *x* or *y* in the above expression. If successive note positions are independent, then the expected value of *R*(1) is zero. If a sharp note is followed by a sharp note (or flat by flat) then the expected value of *R*(1) will be positive. If a sharp (flat) note is followed by a flat (sharp) note then the expected value of *R*(1) is negative. All correlation coefficients lie between +1 and −1. Auto-correlograms are symmetric about *k* = 0 (where *R*(0) = 1.0). A conventional measure for assessing the statistical significance of the correlation coefficients was used (Chatfield, 1975). Here, a significance level of *α* < 0.05 is employed.

### Modeling

In this study we also used models of the shift end-points to help us interpret the correlations derived from the experimental data. These were algorithms that generate an alternating sequence of two end-points on the basis of various assumptions. The note sequences thus generated were subjected to the same analysis applied to the empirical data. Simulated sequences of note positions using these different models were based on the assumption that each movement had a zero mean, normally distributed execution error, an assumption that is not critical.

Two models are used here.

1. *Alternating renewal model*: End-points *P_j_* are chosen independently and alternately from two normal distributions *N* with means *μ*_1_ and *μ*_2_ and standard deviations *σ*_1_ and *σ*_2_.
  $$P_{j}(i+1)=N(\mu_{j},\sigma_{j});j=1,2$$
2. *Wiener process model*. Theoretically, the Wiener process is a continuous random walk process. We have employed a discrete version here, as if sampling from the continuous process. Two simultaneous alternating sequences are generated for comparison with our two-note data sequences. This model is a variant of Wold’s Markov process model (Cox and Lewis, 1966). To generate each successive value in our simulations we take the current value *P(i)* and add a zero-mean, normally distributed random variable.
  $$P_{j}(i+1)=P_{j}(i)+N(\mu_{j},\sigma_{j});j=1,2$$

Model 2 has been well-studied theoretically. Since the values in each of the two sequences are dependent only on their previous values, this is also classified as an alternating Markov (or semi-Markov) process (Einstein, 1956; Bharucha-Reid, 1960; Cox and Lewis, 1966; Iosifescu, 1980). It is also classified as a *martingale* sequence since the expected value of the random variable, *E*[*P*(*I* + 1)] = *P*(*i*). (Bharucha-Reid, 1960) Thus, for two notes with different locations, each with martingale properties, we would have an alternating martingale process. It should be noted that when both note sequences are martingales, Model 2 can also be conceived as an alternating martingale “distance” model. A distance model bases each new end-point on the previous end-point plus or minus some normally distributed distance variable.

While it is a relatively simple matter to simulate a variety of random processes, including classic random walks identifiable by name, it is usually impossible to look at a random—looking data sample and decide if it could be a realization of one of those processes. Usually, we don’t know what features of the data are diagnostic. The Wiener process is a notable exception.

## Results

In this report we present results from subjects shifting alternately between notes B and D, or B and A, on the A-string of the cello. The shift sequences in half the trials were made while using the bow; therefore visual and auditory feedback of the pitch changes accompanying those movements was available. In the other half, subjects were not permitted to use the bow and the subjects’ eyes were closed to prevent any possibility of visual guidance during the movements.

**Figure 1:**
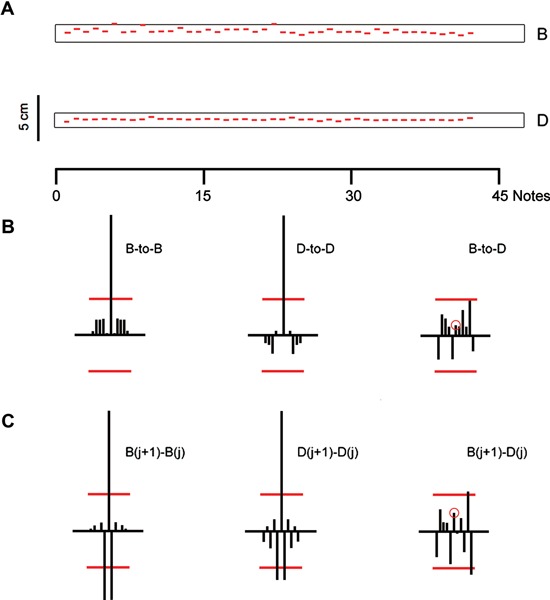
**(A)** Subject shifts between two notes (top trace: note B; lower trace: note D) while using the bow. The two traces show the sequential modal values of finger contact position as measured by a string circuit. The modal location (vertical axis) for each note is shown as a standardized line and plotted horizontally against the note number. Enclosing each note sequence is a “pitch window” notes within that window would not be perceived as having pitch error. **(B)** Serial correlation coefficients for the note sequences shown above. The red lines indicate the values within which coefficients are not significant at the *p* < .05 level. Left: serial auto-correlation coefficients for Note B, lags −5 to +5. Middle: same for note D. Right: cross-serial coefficients with note B the reference note. The red circle indicates the value at *lag* = 0. **(C)** Serial correlation coefficients for the sequential *differences* between notes. Left: Serial auto-correlation coefficients for the sequential *differences* between notes B. Middle: same for note D. Right: cross-serial correlations between sequential note B and note D differences.

In Figure 1A is shown a performance by a subject shifting from note B (61 cm from the cello bridge) to note D (at 51.3 cm). There are 44 notes at each pitch. The shifting distance is not large, and the subject is very close to the true location of the target note most of the time. In a third trace is shown the relatively constant contact distance between B and the next D. Enclosing each note track is a measure we call the “pitch window”. It indicates how much latitude there is in the contact position for each note in order for the note to be perceived, for all but the most discriminating listeners, as having the correct pitch. The pitch window is not the same for any two notes, becoming larger as the distance from the bridge becomes greater.

This example illustrates an important feature of trials using the bow. First, Figure 1A portrays a level of accuracy that would the expected of an experienced performer. We note also the stability of each sequence: no trends are evident. For note B, only two notes are outside the pitch window—just barely. None of the D notes is outside the D pitch window.

For each note sequence in Figure 1 we calculated the standard deviation of contact position. Dividing that value by the size of the pitch window gives us a measure of how the variance of contact position is related to pitch precision. The window for B is larger than that for D, but the variance of contact positions for B is also greater. In percentage terms, the standard deviation of contact points for B is 15% of the window; for D the value is 6%. Thus D has a pitch precision considerably greater than that of B.

In Figure 1B we show the serial auto- and cross-correlation coefficients for the two note sequences shown in Figure 1A. There are no coefficients of statistical significance. The notes within each sequence, while variable and random, are statistically independent of one another. Nor is there dependence between the two notes (at the *p* < .05 level). From a formal point of view these note sequences have the properties of two independent *renewal processes* (Cox and Lewis, 1966).

At the bottom of the figure (Figure 1C) we show a second set of correlation coefficients, this time for the successive *differences* between all notes B, all notes D, and the correlation between the note B and D differences. While the note contact locations are uncorrelated, their serial differences are not. In particular, we note the characteristic significant negative serial autocorrelation coefficient at *lag* = 1. Its expected value is −0.5 (See Wing and Kristofferson, 1973). The patterns of correlations in Figures 1B,C are the basis on which we classify this and other trials as potentially renewal trials.

**Figure 2:**
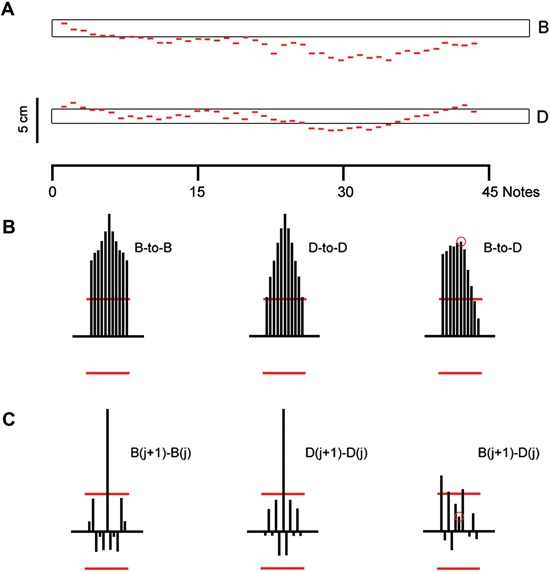
**(A)** Subject shifts between the same two notes as in Figure 1 without the use of bow (and eyes are closed). For reference, the same “pitch window” is again shown. **(B)** Serial correlation coefficients for the note sequences shown above. Left: serial auto-correlation coefficients for Note B, lags −5 to +5. Middle: same for note D. Right: cross-serial coefficients with note B the reference note. **(C)** Serial correlation coefficients for the sequential *differences* between notes. Left: Serial auto-correlation coefficients for the sequential *differences* between notes B. Middle: same for note D. Right: cross-serial correlations between sequential note B and note D differences.

In Figure 2A we show one result when a subject performed the same nominal shifts (B to D) but without using the bow. The pitch windows are shown as before. In contrast with the results in Figure 1, here both B and D notes have a systematic pitch/location error. Both notes also show noticeable *drifts* with changes in drift direction at various points in the trial. This result suggests that *without the bow, this subject had no clear or consistent idea where the target note-positions were located.*

In Figure 2B we show the serial correlations for notes B and D and their cross-serial correlation coefficients. These serial correlations exhibit patterns quite unlike those seen in Figure 1B where the subject used the bow. In particular these correlations are persistently significant over many lags. In Figure 2C we show the serial correlations of the *differences* between sequential notes. The pattern here is similar to the pattern shown in Figure 1B. The absence of any significant correlation suggests that the difference sequences are realizations of renewal processes: statistically, successive *differences* are individually and mutually uncorrelated.

**Figure 3:**
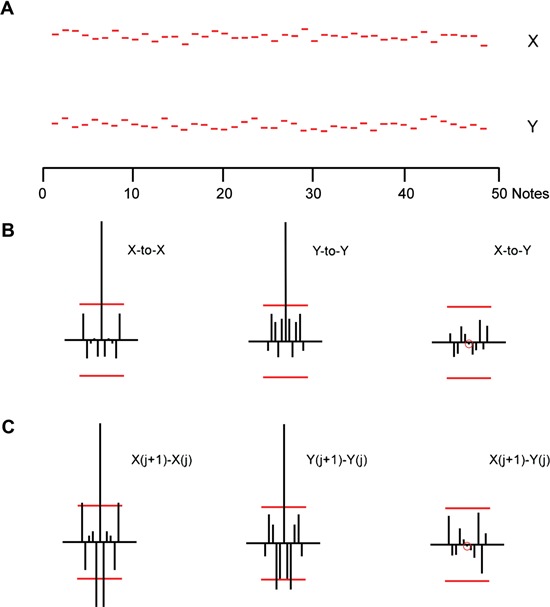
**(A)** Alternating positions of X and Y simulated using a renewal model. **(B)** Serial correlation coefficients for the renewal model sequences shown above. Left: serial auto-correlation coefficients for X. Middle: same for Y. Right: cross-serial coefficients with X the reference. **(C)** Serial correlation coefficients for the sequential *differences* between simulated sequences. Left: Serial auto-correlation coefficients for the sequential *differences* within the renewal sequences X. Middle: Y sequence. Right: cross-serial correlations between sequential X and Y differences. X is the reference.

**Figure 4:**
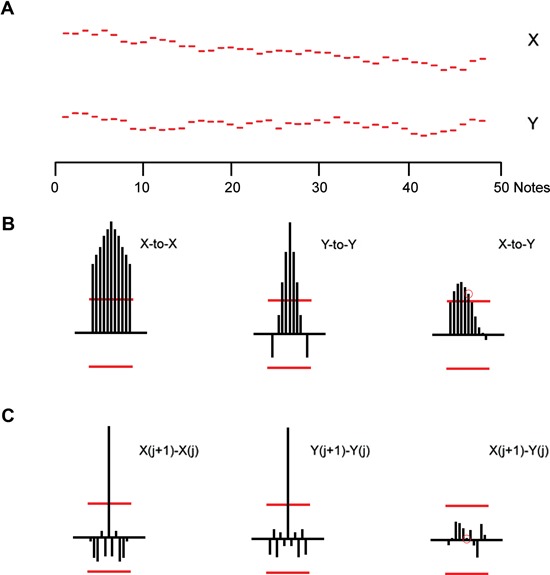
**(A)** Alternating positions of X and Y simulated using a dual Wiener process (martingale) model. **(B)** Serial correlation coefficients for the simulated sequences. Left: serial auto-correlation coefficients for X. Middle: same for Y. Right: cross-serial coefficients with X the reference. **(C)** Serial correlation coefficients for the sequential *differences* between simulated sequences. Left: Left: Serial auto-correlation coefficients for the sequential *differences* within sequence X. Middle: same for Y. Right: cross-serial correlations between sequential X and Y differences. Note X is the reference.

To put these two examples in context, we show, in Figures 3,4 single examples of simulations of two types of random processes: These provide a framework for discussing the behavior of these two subjects in the two performance conditions.

In Figure 3 we show an example of a simulation of a *renewal process* (Methods, Model 1). Each of the “notes” X and Y is generated by adding a normally distributed random variable to the fixed mean values of X and Y. The sequences have expected values X and Y as do the expected values of each note. For our purposes the actual values and their variances are of no interest. Figures 3B,C also shows the serial correlation coefficients of X and Y, their cross-serial correlations; and the correlation coefficients of their sequential differences, as in Figure 1.

In Figure 4 we show an example of a random walk model simulation (Methods, Model 2) that also generates sequences X and Y. Each value *x(i)* is calculated from the *previous value* *x(i*−*1)* to which is added an independent, normally distributed random variable. Whereas in Figure 3 each successive note value is independent of all others, in Figure 4 each note is dependent on (generated from) the previous note value. The expected value of *x(i)* is *x(i*−*1)*. That property of this model makes it a *martingale process*. Since the value of each note is dependent only on the previous note, this is also classified as a *Markov process* (Bharucha-Reid, 1960; Iosifescu, 1980). This sequence may also be thought of as consisting of sequential samplings of a continuous theoretical process known as a *Wiener process* or *Brownian motion process* (Bharucha-Reid, 1960). The serial auto-and cross-correlations of this example are shown in Figure 4B; the serial correlations of the sequential differences are shown in Figure 4C. The *differences* are generated in the simulation algorithm from a sequence of independently chosen, normally distributed random variables; hence they are—theoretically—realizations of a renewal process. The difference correlations (Figure 4C) therefore have a statistical structure equivalent to those shown in Figure 3B. The structure of the sequential correlations (Figures 4B,C) are quite different, but resemble qualitatively, those from the no-bow trial shown in Figures 2B,C.

To what extent are the results in Figures 1,2 representatives, respectively, of bowed and un-bowed trials? Actually, sequences in *both* sets of trials can exhibit the stochastic properties shown in Figures 1,2. That is, some of the bowed sequences have statistical results resembling those in Figure 2; some of the un-bowed sequences resemble those in Figure 1 (see analysis below). Some empirical sequences do not belong clearly to either type, having correlation patterns that are hybrids of the two. These we are presently unable to classify using our models. In the bowing trial shown in Figure 1 none of the autocorrelation coefficients for either note B or D was significant, conforming to the *pattern* associated with a renewal process. Overall, in eight bowed B–D trials, 8 of the 16 sequences showed the Figure 1 pattern; i.e., had no significant autocorrelation coefficients for lags 1–5. In six of these eight trials it was note D that exhibited the renewal pattern. Only two of eight bowed trials showed significant cross-correlations between notes B and D. Figure 1 therefore is representative of many bowed BD trials, though in only two cases did *both* notes exhibit a renewal pattern. For the bowed B–A trials, 5 of 16 usable sequences showed the renewal pattern of Figure 1B; two showed the pattern in Figure 2B.

In the no-bow case, 12 of 13 B–D sequences had significant autocorrelation coefficients such as those shown in Figure 2B. Seven of eight trials had significant cross-serial correlation coefficients. Thus the Figure 2 results are representative of many—but not all—no-bow B–D trials. For the unbowed B–A trials three of five usable sequences showed the Figure 1B (renewal) auto-correlation pattern; two showed the Figure 2B pattern.

Similarly, we can ask if the sequences and correlations shown in Figures 3,4 are representative of repeated simulations of renewal or Wiener process models. No two simulated model sequences were the same, of course, and no correlation sequences were the same, either for the sequences themselves or for the sequences of their differences. But repeated simulations conformed to the basic *pattern* of renewal processes or Wiener processes. To that extent, Figures 3,4 are representative of the basic patterns for each model.

Repeated simulations using the renewal model rarely lead to auto- or cross-correlation coefficients that are statistically significant. The difference correlations are almost invariably characterized by significant negative correlations at *lag* = 1. Repeated simulations using the Wiener model invariably lead to serial auto-correlation coefficients that are significant and persistent, at least when the sample is restricted to 50 values (“notes”) for each sequence. Cross-serial coefficients have patterns that can drift between positive and negative values; rarely are all coefficients without statistical significance at least for lags from −5 to +5. Auto- or cross-correlations coefficients based on the serial *differences* in the Wiener simulation sequences rarely have statistical significance. It follows, then, that there would inevitably be quantitative differences in the details of Figures 1,3 and Figures 2,4; but there are no qualitative differences in pattern for the two trials shown.

## Discussion

We report here four sets of results of cello pitch performance: empirical trials of repeated performance of specific pitch intervals with and without the bow, and models applicable to both sets of the empirical data, each examined consistently from a statistical point of view.

Least surprising are the results obtained when our subjects shift between two notes while using the bow: their intent was to obtain specific auditory outcomes. We have reported earlier on the actions of performers in these conditions (Chen et al., 2006, 2008), noting that small changes in final finger contact location are often made by the performer within the duration of the note. These may be error-correcting movements, possibly in response to perceived errors in pitch. When these note sequences are subjected to statistical analysis, we reach the unexpected conclusion that each of the notes, as defined by their location on the fingerboard, are generally independent of all other notes. In other words there is no indication that any pitch/position errors in either note have observable effects on the pitch/position of subsequent notes. Whatever the effect of any errors, they have been subsumed within each note. These results are summarized by saying that most—though not all—bowed note sequences can be provisionally classified, from their statistical pattern, as *renewal processes*. Behaviorally, these results also indicate that the performer has adopted a consistent strategy within each trial.

On the other hand, when the performer attempts to shift between the same note pairs without using the bow, the precision seen earlier vanishes: the notes no longer lie comfortably within the pitch window of the target note. Even their mean positions may exhibit a systematic error, in addition to drift (not necessarily monotonic) within the sequence. This is perhaps surprising when we recall that pitch and position, and the presumed proprioceptive and tactile cues that accompany each note, have an association established by years of practice. Shifts from B to D (and D to B) using the index finger are commonplace events in performance. Yet without the pitch feedback provided by the bow, our subjects seemed to have only a vague idea of where their contact finger had landed on the fingerboard. Somatic cues alone were inadequate. If performers could hear the pitch equivalent to their contact position they, like all listeners, would immediately perceive the surprisingly large pitch/position errors. Furthermore, in most cases our no-bow data and their serial correlations—utterly unlike the usual note correlations when using the bow—show strong serial correlations within and between the note sequences.

It is important to point out that when deprived of vision, important cues about the spatial location of the performer and the cello are lost. And with the bow no longer even on the string, the performer also loses a potential source of triangulation that may be important for positioning the finger properly on the string.

Of the two note pairs used in our study the B–D shifts are unique: during the shift the hand and arm encounter no distinctive landmarks of the cello body itself, unlike the larger B–A shifts that require arm and hand postural changes to navigate obstacles presented by the instrument. This lack of distinctive landmark features in B–D shifts may make the performer even more dependent on pitch. In several cases no-bow sequences have statistical properties that suggest they are realizations of a random-walk process (Methods, Model 2). In particular, these cases have statistical patterns characteristic of a well-known Markov process related to a theoretical random walk known as the “Wiener process” (Bharucha-Reid, 1960). In our simulated sequences each “note” was based on the previous—but only the previous—note location. Individual realizations (simulations) of such processes are often characterized by local drift, leading to statistically significant and *sustained* serial auto-correlation coefficients, as seen in Figure 4. Though the two sequences in Figure 4 were independently generated, their cross-correlations show sustained and statistically significant coefficients, a result of the fact that such sequences, as in Figure 4, exhibit drifts that partially overlap in time. When our performance data exhibit this feature (Figure 2) we cannot therefore reject the Wiener model as an explanatory summary of the performance. We provide an interpretation of this below.

### Reference Points

Central to most motor control theories is the concept of a “reference point”. The paradoxical issue here is that the key sensory system when acoustic feedback is present is auditory—which does not have a spatial dimension. Both proprioception and vision have spatial aspects; but pitch does not, though the pitch of the note precisely determines the exact spatial location required of the contact between the finger, the string and the fingerboard. When pitch is the reference there must be a mapping from the non-spatial pitch variable to the spatial fingerboard variable. Yet the (hyperbolic) mapping of pitch onto the fingerboard is not simple or linear, and only qualitatively understood by performers. This mapping may require years of practice to achieve accuracy. The fact that note sequences, when auditory feedback is present, are renewal processes indicates that some internal pitch–sense controls central motor behavior, and that the external behavioral realizations do not affect, or corrupt, the internal reference. As noted in a recent study (Brown et al., 2013), “The ways in which pitch and temporal structure in auditory sequences are mapped to the motor system in production remain poorly understood”. This blunt assessment from such a complex study makes it unlikely that we will have any theoretical understanding in the near future of the failures that led to the degradation of our subjects’ no-bow performances. We are not arguing here that vision and proprioception are not important in musical performance; this study shows that they are not by themselves adequate to support the fingering precision that is observed when auditory feedback is also available.

### Behavioral Interpretations of the Data

We assume that the performer, when using the bow, attempts to match the resulting pitch with an internal reference pitch. But what is the performer attempting to do when use of the bow is denied? This requires a change in strategy, or rather a change in reference. But our subjects don’t seem to have a stable absolute spatial reference sense. Instead, our models imply that in many cases the spatial reference is a random variable dependent on the spatial location of the previous note. A “floating reference”, perhaps, leading to a random walk-like succession of executed note locations. (Perhaps in some cases the subject adopts a random inter-note *distance* reference value; in which case, both notes must be martingale-like sequences). In other trials only one note may have the martingale property. For the remainder of our empirical trials the statistical profile seems to be a hybrid of Figures 1,2 correlations; we do not at present have models for these note sequences.

Does this mean that the controller itself is stochastic? It depends. In the case of Figure 2, interpreted by the results of Figure 4, the controller can follow a completely deterministic process, which, because of execution and perception errors, generates a random- walk sequence. The controller can be deterministic but the control reference point is “random” because, in effect, the controller “learns” the previous random reference value and sequentially updates it. It “learns” or “remembers” its own cumulative random execution errors. This is the inevitable result of any controller attempting to reproduce a response already contaminated by execution error. The performer’s strategy or intent may be entirely rational; the controller may be deterministic. The resulting motor behavior is stochastic.

“Execution errors” are assumed to be on the efferent side but perceptual errors, for example a misperception of the hand or finger contact position, are assumed to be on the afferent side. If these errors are additive, the result is still a stationary Markov process—a random walk—assuming the central strategy is unchanged. The subject need not be employing any strategy consciously, but a martingale strategy has one great advantage: the subject need only remember the last perceived location for each note.

Indeed, the performer may be following a similar strategy when using the bow; the central controller may be attempting to return the finger to the previous contact position—or pitch—for a given note. Again, there will be motor execution and proprioceptive errors. If the resulting and possibly error-contaminated pitch/location errors are detected (in relation to the invariant internal pitch reference) and corrected during the time period that note is held (Chen et al., 2008), a renewal process would be expected. Whatever the case, the final contact location, presumably associated with an acceptable pitch, has no influence on subsequent final locations. But if the final pitch/location is memorized by an updated controller, then, in these rare cases, even a bowed sequence could exhibit a Wiener process type of sequence.

In this study we employed a task requiring sequential, alternating movements between two target locations. We studied not only average performance accuracy (see Chen et al., 2006, 2008), but also the serial relations within and between the alternating note location sequences. To our surprise, we found that in the no-bow trials subjects seemed unable to place their fingers at the correct contact positions on the fingerboard, and without auditory feedback seemed unaware of their actual performance. It is possible that the internal pitch the subject imagined was stable and accurate (all trials began with the subject acoustically verifying the correct pitch of note B) but that the processes that normally generate movement to the desired location were compromised. Another possibility is that the internal reference pitches were themselves compromised, though the usual motor processes that translate a desired internal pitch to a spatial location of the finger on the string were intact. Or perhaps there was a combination of these and possibly other malfunctions that degraded the performance.

## Concluding Remarks

Statistical properties of note sequences produced by experienced cellists have been modeled here using simulations of various random processes. Without auditory feedback, even experienced musicians have difficulty shifting alternately and with precision between two notes played with one finger on the same string. The sequence of note positions resembles statistically those resulting from a discrete Wiener process. With auditory feedback, conversely, the same note locations are reached with remarkable precision, an unsurprising result; here the randomness in performance is best modeled as a renewal process. The degradation in performance, when use of the bow is denied, may reflect the distinctly secondary value of proprioceptive and tactile feedback in skilled cellists. This finding gives little support to the common assertion that musicians can produce precision movements in the absence of auditory feedback. Their actual performance, including the random aspects reported here, was unexpected.

## Conflict of interest statement

The authors declare that the research was conducted in the absence of any commercial or financial relationships that could be construed as a potential conflict of interest.
